# 70ProPred: a predictor for discovering sigma70 promoters based on combining multiple features

**DOI:** 10.1186/s12918-018-0570-1

**Published:** 2018-04-24

**Authors:** Wenying He, Cangzhi Jia, Yucong Duan, Quan Zou

**Affiliations:** 1 School of Computer Science and Technology, Tianjin University, Tianjin, 300072 China; 2 Department of Mathematics, Dalian Maritime University, Dalian, 116026 China; 3 College of Information and Technology, Hainan University, Haikou, 570228 China

**Keywords:** sigma70 promoter, PSTNP_SS_, PseEIIP, SVM

## Abstract

**Background:**

Promoter is an important sequence regulation element, which is in charge of gene transcription initiation. In prokaryotes, *σ*^70^ promoters regulate the transcription of most genes. The promoter recognition has been a crucial part of gene structure recognition. It’s also the core issue of constructing gene transcriptional regulation network. With the successfully completion of genome sequencing from an increasing number of microbe species, the accurate identification of *σ*^70^ promoter regions in DNA sequence is not easy.

**Results:**

In order to improve the prediction accuracy of sigma70 promoters in prokaryote, a promoter recognition model 70ProPred was established. In this work, two sequence-based features, including position-specific trinucleotide propensity based on single-stranded characteristic (PSTNPss) and electron-ion potential values for trinucleotides (PseEIIP), were assessed to build the best prediction model. It was found that 79 features of PSTNP_SS_ combined with 64 features of PseEIIP obtained the best performance for sigma70 promoter identification, with a promising accuracy and the Matthews correlation coefficient (MCC) at 95.56% and 0.90, respectively.

**Conclusion:**

The jackknife tests showed that 70ProPred outperforms the existing sigma70 promoter prediction approaches in terms of accuracy and stability. Additionally, this approach can also be extended to predict promoters of other species. In order to facilitate experimental biologists, an online web server for the proposed method was established, which is freely available at http://server.malab.cn/70ProPred/.

**Electronic supplementary material:**

The online version of this article (10.1186/s12918-018-0570-1) contains supplementary material, which is available to authorized users.

## Background

Transcription is strictly regulated and controlled by cis-regulatory DNA elements, which were known as promoters and enhancers. These elements control the level of gene expression and cell fate. Promoters are cis-acting DNA sequences that switch on or off the gene expression. They are generally located upstream of the transcription start sites of genes. In prokaryotes, promoters are identified by RNA polymerase and a related sigma factor [1]. Different *σ* factors interact with well-defined consensus promoter sequences. Each *σ* factor is marked according to its molecular weight. *σ*^70^ is a well-known factor that regulates the transcription of most housekeeping genes in normal circumstances [1]. For *σ*^70^ promoter, two short sequence elements approximately located at around -10 bp and -35 bp nucleotides upstream from the transcription start site (TSS) with consensus TATAAT and TTGACA respectively [2]. It is important to identify the promoters in a genome, because it can help clarify the regulatory mechanism in the genome and explain disease-causing variants within cis-regulatory elements [3, 4]. Meanwhile, it’s a crucial part of gene structure recognition and the core issue of building gene transcriptional regulation network. Man’s understanding of promoter is developing all the time. It’s an area of great concern as people place increasing attention on their importance not only in developmental gene expression but also in environmental response [5, 6].

Due to the rapid development of genome sequencing technology, large-scale data has been generated [7–9], the stable and accurate identification of promoter is an important problem. Because standard laboratory methods are time-consuming and performance overhead costing, bioinformatics technologies with perfect precision represent the ideal alternative for massive fast recognition of promoter.

The *σ*^70^ promoter recognition task is a binary classification task. Feature extraction and classifier design are the key problems in promoter identification technology. In the past 20 years, based on the feature of promoter sequences, a serious of approaches have been developed for detecting promoter region in prokaryotes [10–23]. In 2007, Zhang proposed an algorithm using increment of diversity with quadratic discriminant (IDQD) analysis [21]. Position weight matrix (PWM) is always regarded as a description of the sequence information, but sometimes it gives poor results [22]. After that, Wu proposed an improved Position Weight Matrix (IPWM) [23] in 2011. In the same year, Lin proposed a hybrid method (IPMD), which combines location-related scoring function and diversity increment with improved Mahalanobis Discriminant to predict promoters [16]. The next year, variable-window Z-curve was used for extracting basic features of prokaryotic promoter [14]. Recently, Lin developed an improved Z-curve called ‘multi-window Z-curve’ (PseZNC), which can express the frequency characteristics and three dimensionality characteristics of different length sequences [15], etc. They were mainly focused on the *σ*^70^ promoter recognition. Among these approaches, some typical machine learning algorithms have been used to develop prokaryotic promoter region prediction. Such as, SVM (Support Vector Machine), RF (Random Forest), NB (Naïve Bayes), PLS (Partial Least Square), etc. Although these approaches have contributed to the advancement of promoter recognition, their performance demonstrates that there is a long way to go to predict promoter accurately for the following reasons. (i) Most existing approaches overlooked the correlation of neighboring nucleotides in each position, especially their difference in positive and negative samples. (ii) The local biological and physical properties of DNA may have a certain relationship with the promoters, which plays an important role in identifying them but were utterly ignored. (iii) Few web-servers were provided as the predictors, and hence their usage is quite limited [24].

In current research, we exploited a new bioinformatics tool called 70ProPred, to predict *σ*^70^ promoter through a combination of position-specific trinucleotide propensity (PSTNP) and electron-ion interaction pseudopotentials (EIIPs) of nucleotides. Finally, based on the results analysis of jackknife test, 70ProPred significantly outperforms existing prediction models, and should be useful for identifying *σ*^70^promoter.

## Methods

**Fig. 1:**
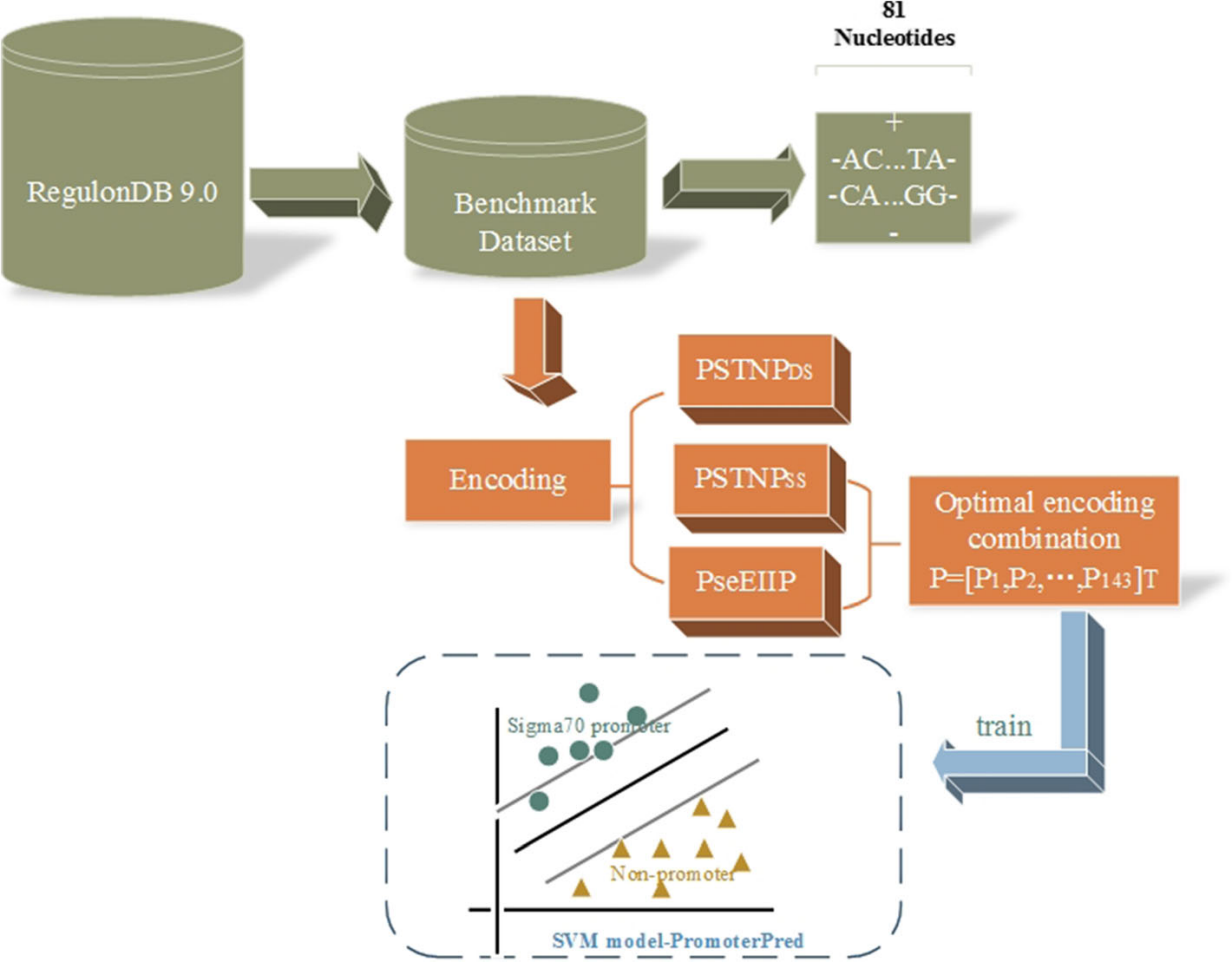
Overview of the proposed 70ProPred predictor. The diagram mainly contains datasets, sequence descriptors and 70ProPred prediction system. The optimal encoding combination PSTNP_SS_ and PseEIIP are used as the input to train a SVM classifier. After optimization of the SVM parameters, the best SVM model is constructed based on the jackknife performance

Briefly, 70ProPred is a prediction model based on support vector machine (SVM), which was built by PSTNP_SS_ and PseEIIP sequence coding strategies. An outline of the computational framework of 70ProPred predictor is shown in Fig. 1.

### Datasets

In the present study, we used the latest datasets in both [15] and [16]. A total of 741 *σ*^70^ promoter samples were selected from the *E.coli* K-12 genome, which have been verified by experiments and downloaded from the RegulonDB9.0 (http://regulondb.ccg.unam.mx/) [25]. The promoter region [TSS-60…TSS + 20] (the locus of TSS is 0) were prepared as positive samples with a length of 81 bp.

As there are not enough experimental confirmed negative sequences, negative samples are collected from both coding and non-coding regions. In simple terms, the benchmark dataset S used in this study can be expressed as:

1 $$ \left\{\begin{array}{c}S={S}^{+}\cup {S}^{-}\\ {}{S}^{-}={S}_{\mathrm{coding}}^{-}\cup {S}_{\mathrm{non}\hbox{-} \mathrm{coding}}^{-}\end{array}\right. $$

Where *S*^+^ contains 741 *σ*^70^ promoter samples, *S*^−^ contains 1400 non-promoter samples, ${S}_{\mathrm{coding}}^{-}$ contains 700 coding sequences, ${S}_{\mathrm{non}\hbox{-} \mathrm{coding}}^{-}$ contains 700 convergent intergenic sequences. Each sample contains 81 nucleotides, which is selected by a sliding window. Additionally, symbol ∪ means union.

### Analysis of *σ*^70^ promoter samples for conserved motif composition

**Fig. 2:**
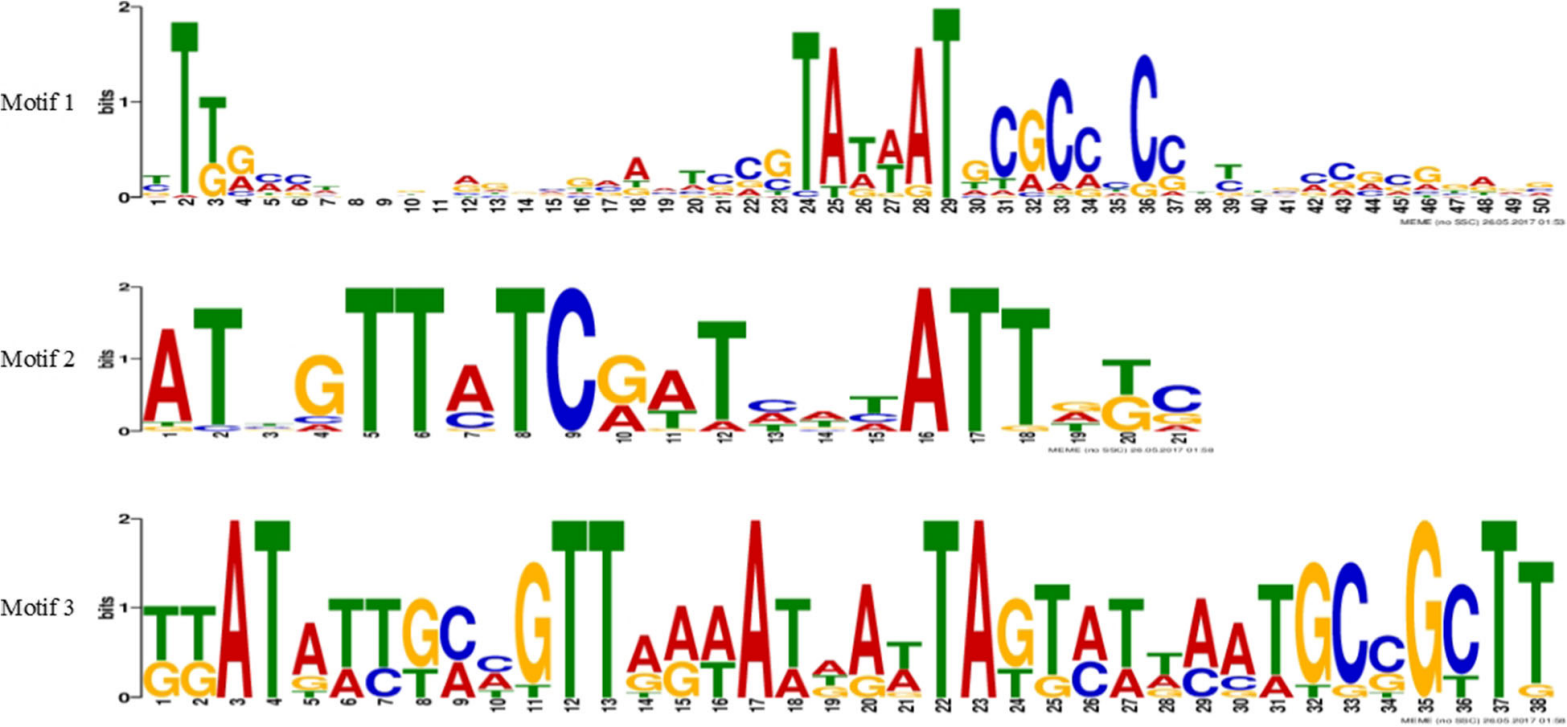
Motif of *σ*^70^ promoter samples as found by the MEME system. The corresponding three-motif logos as visualized for *σ*^70^ promoter samples (details in Table 1)

The MEME Suite is designed to screen common sequence motifs from a set of sequences (amino acid or nucleotide). A motif can be assumed to be a conservative sequence pattern that repeats itself over a set of related sequences [26]. MEME is a useful sequence analysis tool that can rapidly detect new, non-gapped motifs for biological sequence data (protein, DNA and RNA) [27]. Then, we applied this tool to analyze the main motifs of *σ*^70^ promoter samples and found that only a small part of these samples which have corresponding motifs in Fig. 2. The maximum number of motif was set to 3 and the remaining arguments were set to default.

Although phylogenetic foot printing takes the advantage of relatively conservative of motifs between species [28], these motifs are short and not complete species [29, 30]. For example, in Table 1, the number of sites contributing to the construction of motif 1 only 47, which may result in a great deal of false positive results. Therefore, it would be practicable to turn to the machine learning-based methods and has been proved to be effective in many fields [26, 31–46].

**Table 1:** Conserved motifs of *σ*^70^ promoter samples identified by the MEME system

Motif	Width	Best possible match	Sites count
1	50	YTKRMMWNNBNRGNVGVAMTSCGTATWATGCGCCYCCNYBVMCVCGKRVV	47
2	21	ATBGTTATCRATHWHATTDKC	20
3	38	KKATATTGMHGTTRRWATDAWTAGTMTWAATGCSGCTT	10

### Feature vector construction

In order to get more useful information from the sequence, we extracted two kinds of features. Position-specific tendencies of trinucleotide (PSTNPss or PSTNP_DS_) was adopted to reveal the differences in the distribution of all positive and negative samples between trinucleotide. While the electron-ion interaction pseudopotentials (PseEIIP) were adopted to represent the interaction of trinucleotides.

### Position-specific trinucleotide propensity based on single-stranded or double-stranded characteristic of DNA (PSTNP_SS_/PSTNP_DS_)

The recently proposed position-specific theory has been successfully applied to numerous fields of bioinformatics, such as identifying protein S-nitrosylation sites [47], hydroxyproline and hydroxylysine sites [48], DNA replication origin sites [49], Enhancer [41], etc. Besides, a series of studies have shown that the trinucleotides composition was effective in recognition of promoter [28, 50–52]. Inspired by the above studies, we presented a novel feature extraction strategy, which combined position-specific trinucleotide property (PSTNP) with the K-mer nucleotide composition information to predict *σ*^70^ promoter. A brief account of feature extraction is as follows.

Let *S* represents a sample which was consisted of A, G, C, and T, i.e.

2 $$ S={N}_1{N}_2{N}_3\cdots {N}_L $$

In which *L* means the length of the sample and *L* = 81, and

3 $$ {N}_i\in \left\{\mathrm{A},\mathrm{C},\mathrm{G},\mathrm{T}\right\}\left(i=1,2,\dots, L\right) $$

represents the *i*-th position of corresponding nucleotide in the sequence.

### PSTNP_SS_

Feature PSTNP_SS_ using a statistical strategy based on single-stranded characteristics of DNA. There are 4^3^ = 64trinucleotides: AAA, AAC, AAG, ..., TTT. So, for an 81 bp sample, its details of the trinucleotides position specificity can be expressed by the following 64 × 79 matrix [41]:

4 $$ Z=\left[\begin{array}{cccc}{z}_{1,1}& {z}_{1,2}& \cdots & {z}_{1,79}\\ {}{z}_{2,1}& {z}_{2,2}& \cdots & {z}_{2,79}\\ {}\vdots & \vdots & \cdots & \vdots \\ {}{z}_{64,1}& {z}_{64,2}& \cdots & {z}_{64,79}\end{array}\right] $$

where the variable

5 $$ {\displaystyle \begin{array}{l}{z}_{i,j}={F}^{+}\left(3{mer}_i|j\right)-{F}^{-}\left(3{mer}_i|j\right)\kern0.4em \\ {}\kern1.6em \left(i=1,2,\dots, 64;j=1,2,...\mathrm{79}\right)\end{array}} $$

*F*^+^(3*mer*_*i*_| *j*) and *F*^−^(3*mer*_*i*_| *j*) denote the frequency of the *i*-th trinucleotide (3mer_*i*_) at the *j*-th position appear in the positive (*S*^+^) and negative (*S*^*−*^) data sets, respectively. In the formula, 3mer_1_ equals AAA,3mer_2_ equals AAC, …, 3mer_64_ equals TTT.

Therefore, the sample of Eq.2 can be expressed as:

6 $$ \mathrm{S}={\left[{\phi}_1,{\phi}_2,\dots, {\phi}_u,\dots, {\phi}_{79}\right]}^T $$

where T is the operator of transpose and *ϕ*_*u*_ was defined as follows:

7 $$ {\phi}_u=\left\{\begin{array}{c}{z}_{1,u},\kern0.8000001em \mathrm{when}\kern0.2em {\mathrm{N}}_u{\mathrm{N}}_{u+1}{\mathrm{N}}_{u+2}=\mathrm{AAA}\\ {}{z}_{2,u},\kern0.8000001em \mathrm{when}\kern0.2em {\mathrm{N}}_u{\mathrm{N}}_{u+1}{\mathrm{N}}_{u+2}=\mathrm{AAC}\\ {}{z}_{3,u},\kern0.8000001em \mathrm{when}\kern0.2em {\mathrm{N}}_u{\mathrm{N}}_{u+1}{\mathrm{N}}_{u+2}=\mathrm{AAG}\\ {}\begin{array}{cc}\begin{array}{cc}\begin{array}{cc}\kern0.1em \begin{array}{cc}\vdots & \end{array}& \end{array}& \vdots \end{array}& \end{array}\kern1.00em \vdots \\ {}{z}_{64,u},\kern0.7em \mathrm{when}\kern0.2em {\mathrm{N}}_u{\mathrm{N}}_{u+1}{\mathrm{N}}_{u+2}=\mathrm{TTT}\end{array}\right.\kern1.1em \left(1\le u\le 79\right)\kern0.3em $$

### PSTNP_DS_

**Fig. 3:**
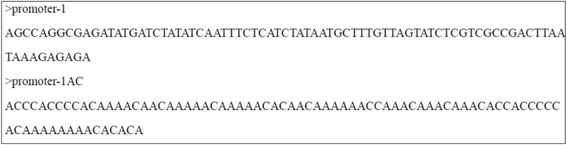
promoter-1 converted into promoter-1 AC

Feature PSTNP_DS_ using a statistical strategy based on double-stranded characteristics of DNA according to complementary base pairing, so they have more evident statistical features. At this point, we deem A and T as identical, the same to C and G. Thus, for every sample, it can be converted into a sequence contained A and T only. As shown in Fig. 3, promoter-1 converted into promoter-1 AC.

So, there are 2^3^ = 8 trinucleotides: AAA, AAC, ..., CCC. Therefore, for an 81 bp sample, its details of the trinucleotides position specificity can be expressed by the following 8 × 79 matrix:

8 $$ {Z}^{\hbox{'}}=\left[\begin{array}{cccc}{z^{\hbox{'}}}_{1,1}& {z^{\hbox{'}}}_{1,2}& \cdots & {z^{\hbox{'}}}_{1,79}\\ {}{z^{\hbox{'}}}_{2,1}& {z^{\hbox{'}}}_{2,2}& \cdots & {z^{\hbox{'}}}_{2,79}\\ {}\vdots & \vdots & \cdots & \vdots \\ {}{z^{\hbox{'}}}_{8,1}& {z^{\hbox{'}}}_{8,2}& \cdots & {z^{\hbox{'}}}_{8,79}\end{array}\right] $$

where the variable

9 $$ {\displaystyle \begin{array}{l}{z^{\hbox{'}}}_{i,j}={F}^{+}\left(3{mer}_i|j\right)-{F}^{-}\left(3{mer}_i|j\right)\kern0.4em \\ {}\kern1.6em \left(i=1,2,\dots, 8;j=1,2,...\mathrm{79}\right)\end{array}} $$

*F*^+^(3*mer*_*i*_| *j*) and *F*^−^(3*mer*_*i*_| *j*) denote the frequency of the *i*-th trinucleotide (3mer_*i*_) at the *j*-th position appear in the positive (*S*^+^) and negative (*S*^*−*^) data sets, respectively. In the formula, 3mer_1_ equals AAA,3mer_2_ equals AAC, …, 3mer_8_ equals CCC.

Therefore, the sample of Eq.2 can be expressed as:

10 $$ {\mathrm{S}}^{\hbox{'}}={\left[{\phi}_1^{\hbox{'}},{\phi}_2^{\hbox{'}},\dots, {\phi}_u^{\hbox{'}},\dots, {\phi}_{79}^{\hbox{'}}\right]}^T $$

where T is the operator of transpose and *ϕ*^'^_*u*_ was defined as follows:

11 $$ {\phi^{\hbox{'}}}_u=\left\{\begin{array}{c}{z^{\hbox{'}}}_{1,u},\kern0.8000001em \mathrm{when}\kern0.2em {\mathrm{N}}_u{\mathrm{N}}_{u+1}{\mathrm{N}}_{u+2}=\mathrm{AAA}\\ {}{z^{\hbox{'}}}_{2,u},\kern0.8000001em \mathrm{when}\kern0.2em {\mathrm{N}}_u{\mathrm{N}}_{u+1}{\mathrm{N}}_{u+2}=\mathrm{AAC}\\ {}{z^{\hbox{'}}}_{3,u},\kern0.8000001em \mathrm{when}\kern0.2em {\mathrm{N}}_u{\mathrm{N}}_{u+1}{\mathrm{N}}_{u+2}=\mathrm{ACA}\\ {}\begin{array}{cc}\begin{array}{cc}\begin{array}{cc}\begin{array}{cc}\kern0.1em \begin{array}{cc}\vdots & \end{array}& \end{array}& \vdots \end{array}& \end{array}& \end{array}\kern0.5em \vdots \\ {}{z^{\hbox{'}}}_{8,u},\kern0.7em \mathrm{when}\kern0.2em {\mathrm{N}}_u{\mathrm{N}}_{u+1}{\mathrm{N}}_{u+2}=\mathrm{CCC}\end{array}\right.\kern1.1em \left(1\le u\le 79\right)\kern0.3em $$

### Electron-ion interaction pseudopotentials of trinucleotide (PseEIIP)

Nair [53] came up with electron-ion interaction pseudopotentials (EIIP) value of nucleotides A, G, C, T. The EIIP value based methods have been shown effective through previous studies, such as the recognition of gene F56F11.4, prediction of the cystic-fibrosis gene [54], recognition of enhancer [41], and so on [55, 56].

The electron-ion interaction pseudopotentials value for the nucleotides [53] are shown in Table 2. We let *EIIP*_*A*_, *EIIP*_*T*_, *EIIP*_*G*_, and *EIIP*_*C*_ denote the EIIP values of nucleotides A, T, G and C, respectively. Then, we employed the mean EIIP value of trinucleotides in each sample to construct feature vector, which can be formulated as:

12 $$ V=\left[{EIIP}_{AAA}\cdot {f}_{AAA},{EIIP}_{AAC}\cdot {f}_{AAC},\dots, {EIIP}_{TTT}\cdot {f}_{TTT}\right] $$

where *f*_*xyz*_ equal to the normalized frequency of the *i*-th trinucleotide (3mer_*i*_), *EIIP*_*xyz*_ *= EIIP*_*x*_ + *EIIP*_*y*_ + *EIIP*_*z*_ expresses the EIIP value of one trinucleotide and X, Y, Z∈ [A, C, G, T]. Obviously, the dimension of vector V is 64.

**Table 2:** EIIP values of nucleotides

Nucleotide	EIIP(Ry)
A	0.1260
T	0.1335
G	0.0806
C	0.1340

### Model building and parameter selection

SVM classification algorithm plays a significant role in some areas of bioinformatics [18, 36, 40, 57]. In this work, SVM was implemented using the LIBSVM packet [58] to build models and execute predictions. The radial basis function (RBF) was selected as the kernel function. At the same time, penalty parameter C and kernel parameter γ were optimized using SVMcg in the LIBSVM package. The final parameters C **=** 22.6274 and γ = 2.8284 were selected for the prediction of *σ*^70^ promoters and non-promoters.

The jackknife test is regarded as a unique random test that can produce the unique result for a given dataset [59]. Therefore, all these parameters were optimized through jackknife test.

In order to evaluate the predictive performance of the model, four metrics are calculated: sensitivity (Sn), specificity (Sp), accuracy (Acc) and MCC:

13 $$ Sn=\frac{TP}{TP+ FN} $$

14 $$ SP=\frac{TN}{TN+ FP} $$

15 $$ Acc=\frac{TP+ TN}{TP+ TN+ FP+ FN} $$

16 $$ MCC=\frac{TP\times TN- FP\times FN}{\kern0em \sqrt{\left( TP+ FP\right)\left( TP+ FN\right)\left( TN+ FP\right)\left( TN+ FN\right)}} $$

In the formula, TP, TN, FP and FN represent the numbers of true positives (accurately predicted *σ*^70^ promoters), true negatives (accurately predicted non-promoters), false positives (falsely predicted *σ*^70^ promoters) and false negatives (falsely predicted non-promoters).

## Results and discussion

### Prediction of *σ*^70^ promoter using only PSTNP

PSTNP was first proposed for predicting enhancer [41]. The obvious advantage of this approach is that the feature vectors are encoded in a way that contains information from all training samples. In this work, the ability of PSTNP_SS_ and PSTNP_DS_ to discriminate *σ*^70^ promoter and non-promoter were first declared by jackknife test (Table 3). For *σ*^70^ promoter, the PSTNP_DS_ model obtained a good performance, reaching at 75.98% sensitivity, 88.57% specificity, 84.21% accuracy and the 0.6493 of MCC value, while the PSTNP_SS_ model obtained a Sn of 90.82%, a Sp of 96.57%, an Acc of 94.58% and a MCC of 0.8797.

**Table 3:** Jackknife test performance of PSTNP_SS_ and PSTNP_DS_

Features	Sn (%)	Sp (%)	Acc (%)	MCC	SVM
PSTNP_SS_ (79)	90.82	96.57	94.58	0.8797	-c 22.6274 -g 1.4142
PSTNP_DS_ (79)	75.98	88.57	84.21	0.6493	-c 1.4142 -g 2.8284 -w1 1.2 -w-1 1

**Fig. 4:**
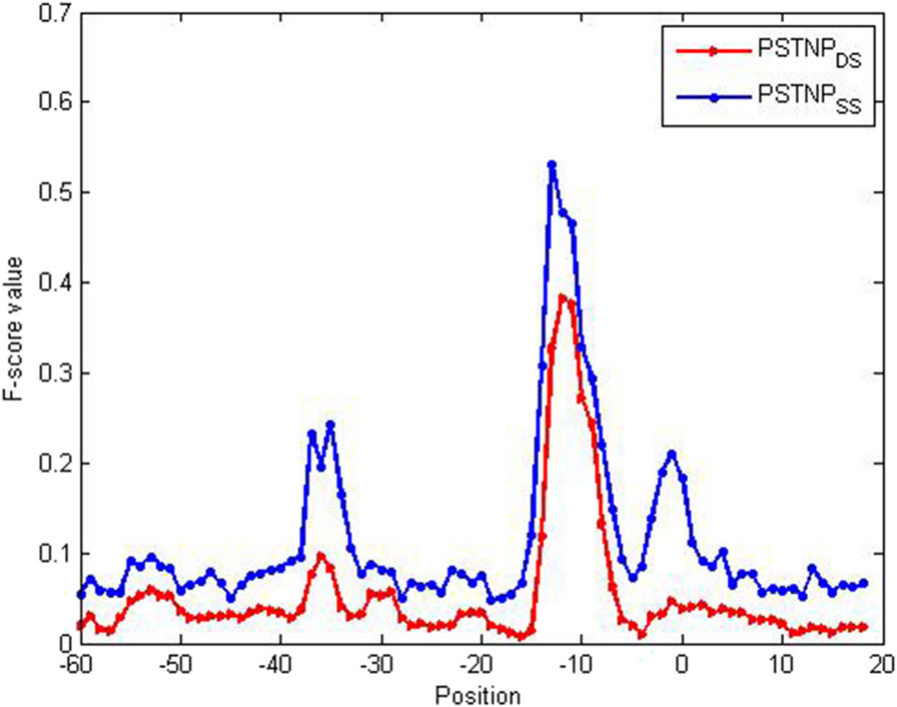
*F-score* value of trinucleotides in both PSTNP_SS_ and PSTNP_DS_

A comparative figure (Fig. 4) with *F-value* of trinucleotides in different position also declared the difference in forecast results.

**Fig. 5:**
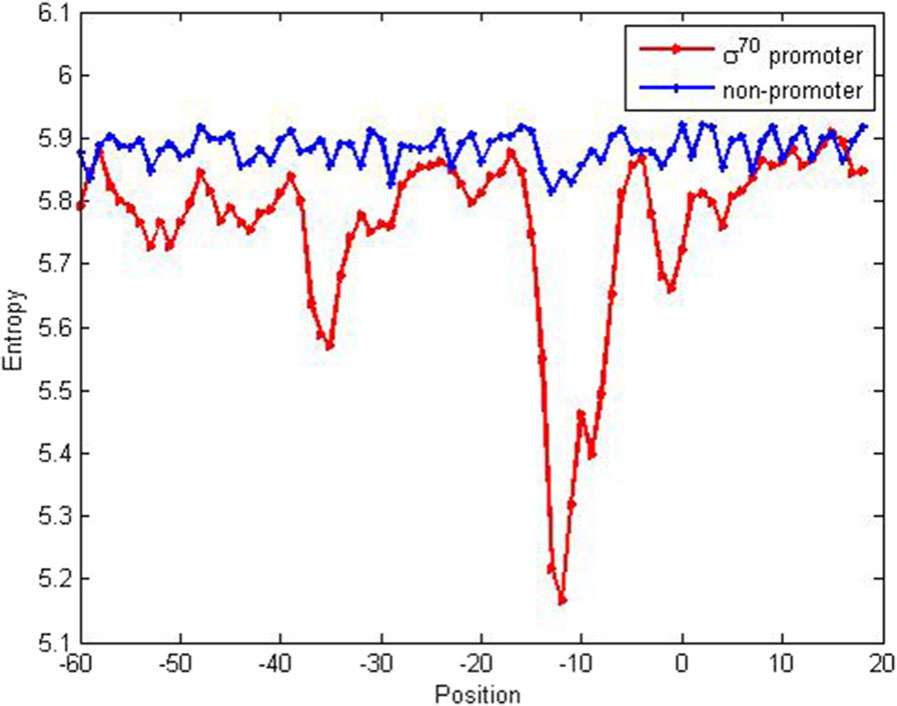
Entropy of trinucleotide in the *σ*^70^ promoter and non-promoter

Furthermore, we used the Entropy (Additional file 1) [23, 60] to demonstrate the conservative sites of trinucleotides property in *σ*^70^ promoter. In order to comparing, the calculated entropy values for the trinucleotide of the *σ*^70^ promoter and non-promoter are shown in Fig. 5. Obviously, the lower entropy, the more conservative the position is. From this figure, we can see that in *σ*^70^ promoter most sites are obviously of lower entropy values compared with non-promoter. It may prove that PSTNP_SS_ feature extraction agreed well with the previous prediction that in different positions the trinucleotide is conservative in *σ*^70^promoter.

From all above, the composition of trinucleotide which based on single-stranded characteristics of DNA contributes to the detection of *σ*^70^ promoter. This result indicated that the PSTNP_SS_ model performs better than the PSTNP_DS_ model in *σ*^70^ promoter prediction. Consequently, the training model optimized on the basis of the feature PSTNP_SS_.

### Improving performance by incorporating PseEIIP

Because the physicochemical property indexes of nucleotides affect the recognition of promoter, incorporating the sample’s average energy of delocalized electrons (EIIP), especially, the EIIP value of trinucleotides with PSTNP_SS_ might boost the performance of the training model, the prediction results are listed in Table 4.

**Table 4:** Performances of our model on the jackknife test

Features	Sn (%)	Sp (%)	Acc (%)	MCC
PSTNP_SS_ (79)	90.82	96.57	94.58	0.8797
PSTNP_SS_ (79) + PseEIIP (64)	93.12	96.86	95.56	0.9018

Eventually, the prediction model was established using the PSTNP_SS_ + PseEIIP feature extraction methods combined with the SVM classifier (cost parameter –c 22.6274, −g 2.8284) to predict *σ*^70^promoter.

**Fig. 6:**
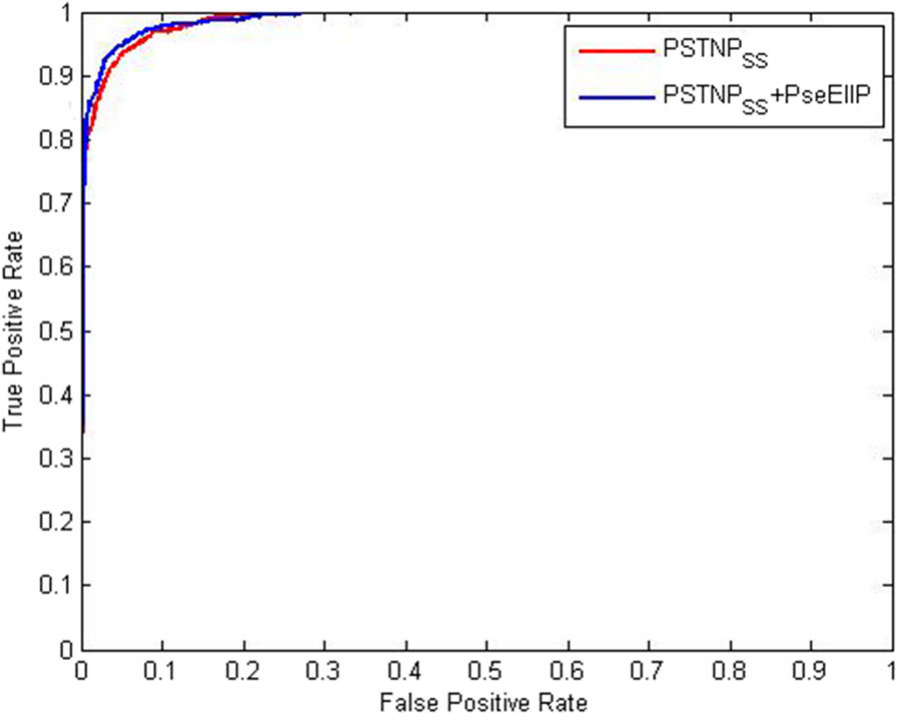
The ROC curves to assess the predictive performance based on different sequences encoding schemes for *σ*^70^promoter

In order to gauge the predictive performance of training model, the ROC curve and the area under the ROC curve (AUC) were adopted. The AUC value the 70ProPred model was 0.990 (Fig. 6).

**Fig. 7:**
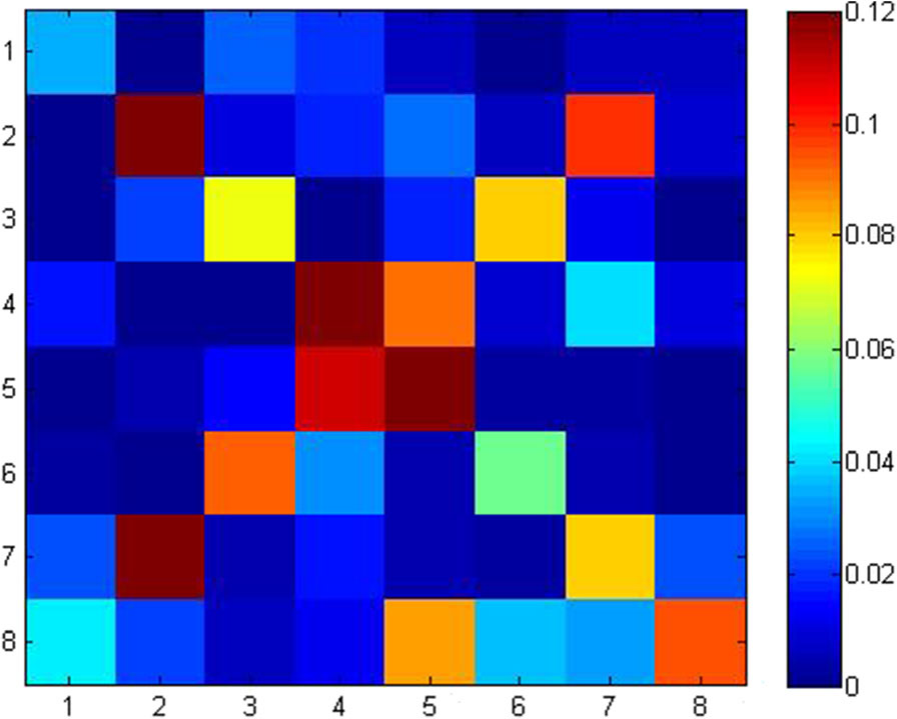
A heat map for the *F-score* values of the 64 trinucleotides with different EIIP values. The blue boxes indicate the features with a lower effect for recognition of the *σ*^70^ promoter, while the red boxes indicate the features that are useful for the recognition of the *σ*^70^promoter

Furthermore, we constructed a heat map to visually show the distribution of feature PseEIIP in positive dataset, as shown in Fig. 7. Each hotspot in the heat map corresponds to a unique trinucleotide; for instance, hotspot (1, 1) corresponds to triplet AAA. For more detailed information on the heat map, please see Additional file 2: Table S1. Red squares are positively associated with recognition ability.

### Comparison of the performance between SVM and other classifiers

In order to select a better classifier to identify *σ*^70^ promoter, we analyzed the performances of KNN [61], NB [62], RF [63], EB [64], LibD3C [65], GBDT [66] and SVM, which are the most widely used classification algorithms in bioinformatics. Since it is known that the number of neighbors has an impact on the performance of KNN algorithm and the tree number has an impact on the performance of RF algorithm, the optimal parameters of KNN and RF are searched in the study, as shown in Additional file 3: Table S2 and Additional file 4: Table S3.

The performances for the above classifiers in the jackknife test are shown in Table 5. The results indicate that SVM performs better than the other classifiers with the highest MCC value 0.9018.

**Table 5:** Comparison of different classifiers for identifying *σ*^70^promoter

Classifier	Sn (%)	Sp (%)	Acc (%)	MCC
KNN (8)	87.04	96.21	93.04	0.8450
Naïve Bayes	91.90	89.00	90.00.	0.7891
Random Forest (200)	85.29	97.79	93.46	0.8548
Ensembles for Boosting (200)	89.88	95.29	93.41	0.8541
LibD3C	77.33	87.57	84.03	0.6478
GBDT	86.50	96.14	92.81	0.8397
SVM	93.12	96.86	95.56	0.9018

### Comparison of 70ProPred with other *σ*^70^ promoter prediction methods

The 5-fold cross-validation results achieved by Z-curve [14], PseZNC [15], IPMD [16], PSTNP_DS_ and 70ProPred on the benchmark dataset are listed in Table 6. Their marked difference is the feature extraction strategy. Therefore, the advantage of 70ProPred superior to other methods is mainly due to the combination of PSTNP_SS_ and PseEIIP coding strategy. PSTNP_SS_ employs primary sequence information of trinucleotides, and PseEIIP is closely related to the physicalchemical property of DNA sample. As shown in Table 3, based on the PSTNP_SS_ feature only, the prediction result of 70ProPred was significantly higher than the other methods. The performance has been further improved by adding the feature PseEIIP, as shown in Table 4, especially in sensitivity (Sn). Taken together, the application of feature PSTNP_SS_ and feature PseEIIP achieved a good performance in the prediction of *σ*^70^promoter.

**Table 6:** performances of our model, Z-curve, PSTNP_DS_, PseZNC and IPMD on 5-fold cross-validation

Methods	Sn (%)	Sp (%)	Acc (%)	MCC	AUC
Z-curve	74.6	79.5	77.8	0.527	0.848
PSTNP_DS_	75.9	88.0	83.8	0.641	0.911
PseZNC	80.3	86.8	84.5	0.663	0.909
IPMD	82.4	90.7	87.9	0.731	–
70ProPred	92.4	96.9	95.3	0.897	0.990

The results in Table 6 also show that the PSTNP_DS_-based model performs better than the multi-window Z-curve-based method. It can also be a supplement to the present methods for predicting other DNA related predictions.

## Conclusions

The 70ProPred is a new bioinformatics tool for predicting *σ*^70^ promoter. This tool uses the feature extraction methods of PSTNP_SS_ and PseEIIP. The combination of features and SVM could achieve an overall MCC value of 0.90. Compared to other *σ*^70^ promoter prediction models, 70ProPred produced better results. Although this method shows good performance in *σ*^70^ promoter prediction, there is still room to improve prediction performance due to the following reasons. (i) Since structural information is a supplementary to sequence information, the future work may build a model combine with the two aspects. (ii) The feature selection algorithms can be used to delete the redundant features to improve the prediction model. (iii) More species of promoters should be adopted to estimate the performance of 70ProPred method. In conclusion, our future work is to extend this method to other species promoter region prediction. We suspect that our feature extraction methods is not only suitable for identifying promoter, but also for other bioinformatics sequence classification tasks.

### Availability

The web-server for 70ProPred has been established. It is now freely available to all interested users at http://server.malab.cn/70ProPred/. All the data sets used in this study can also be download on the website.

## Additional files

Additional file 1: Entropy. (DOC 94 kb)

Additional file 2: **Table S1.** Rules of composition of heat maps. (DOC 41 kb)

Additional file 3: **Table S2.** Comparison prediction results of different k neighbors. (DOC 47 kb)

Additional file 4: **Table S3.** Comparison prediction results of different nTrees. (DOC 42 kb)

## Abbreviations

AUC: Area Under the ROC Curve
EB: Ensembles for Boosting
GBDT: Gradient Boosting Decision Tree
KNN: k-Nearest Neighbor
MCC: Matthew’s Correlation Coefficient
NB: Naïve Bayes
PseEIIP: Electron-ion interaction pseudopotentials of nucleotides
PSTNP_DS_: Position-specific trinuclotide propensity based on double-stranded characteristic of DNA
PSTNPss: Position-specific trinuclotide propensity based on single-stranded characteristic of DNA
RF: Random Forest
