# Supplementary material for: 70ProPred: a predictor for discovering sigma70 promoters based on combining multiple features
Source: BMC Syst Biol. 2018 Apr 24;12(Suppl 4):44. doi: 10.1186/s12918-018-0570-1 (PMC5998878; doi:10.1186/s12918-018-0570-1)
Supplement: Supplementary file 1 — Entropy. (DOC 94 kb) [file 12918_2018_570_MOESM1_ESM.doc]

**Entropy**

In this paper, we propose to use the Entropy defined in[23, 60] to reveal the trinucleotide’s conservative position in $\sigma^{70}$promoter：


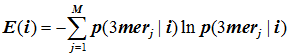


Where$p(3mer_{j}|i)$denotes the occurrence frequency of the *j*-th trinucleotide (3mer*_j_*) at the *i*-th position in the positive/negative dataset. M is the number of all trinucleotides. Obviously, the position with low entropy can be considered conservation. Additionally, to avoid $p(3mer_{j}|i)$taking a value of 0 and hence causing a numerical problem in the logarithm function; we adopt

$$p^{'}(3mer_{j}|i)\text{=}\frac{n(3mer_{j}|i)+s(3mer_{j}|i)}{N_{i}+\sum_{j} s(3mer_{j}|i)}$$

as the estimated occurrence frequency $p(3mer_{j}|i)$. Where $n(3mer_{j}|i)$and$N_{i}$are respectively the counts that $3mer_{j}$appears at the *i*-th position and the total number of sequences in corresponding dataset. $s(3mer_{j}|i)$is the pseudo-count function given by

$s(3mer_{j}|i)\text{=}\frac{\sqrt{N_{i}}}{M}$.

To comparison, the entropy values calculated on the trinucleotide of the $\sigma^{70}$promoter and non-promoter are shown in Figure 5.


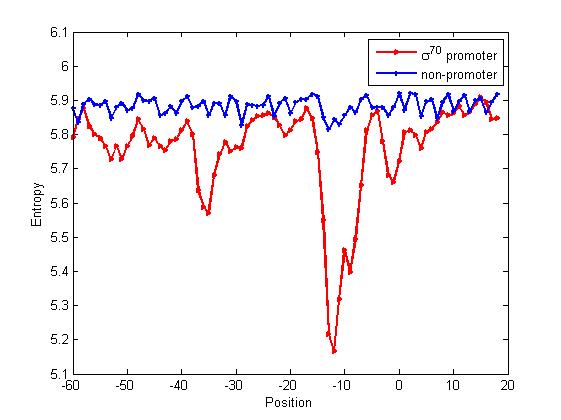


**Figure 5** **The entropy of trinucleotide in the$\sigma^{70}$promoter and non-promoter.**

Obviously, the site with low entropy can be considered conservative in the sequence. From this figure, we can see that in $\sigma^{70}$promoter most sites are obviously of relatively low entropy values compared with non-promoter. It may prove that PSTNP_SS_ feature extraction coincide well with the original prediction that in different regions the trinucleotide is conservative in $\sigma^{70}$promoter.
