# Supplementary material for: 70ProPred: a predictor for discovering sigma70 promoters based on combining multiple features
Source: BMC Syst Biol. 2018 Apr 24;12(Suppl 4):44. doi: 10.1186/s12918-018-0570-1 (PMC5998878; doi:10.1186/s12918-018-0570-1)
Supplement: Supplementary file 2 — Table S1. Rules of composition of heat maps. (DOC 41 kb) [file 12918_2018_570_MOESM2_ESM.doc]

**Table S1** **Rules of composition of heat maps (Figure 7)**

| AAA | AAC | ACA | ACC | CAA | CAC | CCA | CCC |
| --- | --- | --- | --- | --- | --- | --- | --- |
| AAG | AAT | ACG | ACT | CAG | CAT | CCG | CCT |
| AGA | AGC | ATA | ATC | CGA | CGA | CTA | CTC |
| AGG | AGT | ATG | ATT | CGG | CGT | CTG | CTT |
| GAA | GAC | GCA | GCC | TAA | TAC | TCA | TCC |
| GAG | GAT | GCG | GCT | TAG | TAT | TCG | TCT |
| GGA | GGC | GTA | GTC | TGA | TGC | TTA | TTC |
| GGG | GGT | GTG | GTT | TGG | TGT | TTG | TTT |
