# Supplementary material for: 70ProPred: a predictor for discovering sigma70 promoters based on combining multiple features
Source: BMC Syst Biol. 2018 Apr 24;12(Suppl 4):44. doi: 10.1186/s12918-018-0570-1 (PMC5998878; doi:10.1186/s12918-018-0570-1)
Supplement: Supplementary file 3 — Table S2. Comparison prediction results of different k neighbors. (DOC 47 kb) [file 12918_2018_570_MOESM3_ESM.doc]

**Table S2 Comparison prediction results of different k neighbors**

| k | Sn (%) | Sp (%) | Acc (%) | MCC |
| --- | --- | --- | --- | --- |
| 5 | 85.56 | 96.57 | 92.76 | 0.8386 |
| 7 | 84.48 | 96.93 | 92.62 | 0.8356 |
| **8** | **87.04** | **96.21** | **93.04** | **0.8450** |
| 9 | 83.27 | 97.29 | 92.43 | 0.8316 |
| 10 | 86.10 | 96.43 | 92.85 | 0.8407 |
| 15 | 81.78 | 97.36 | 91.97 | 0.8213 |
| 20 | 82.05 | 97.57 | 92.20 | 0.8267 |
| 25 | 81.11 | 97.71 | 91.97 | 0.8217 |
