# Supplementary material for: 70ProPred: a predictor for discovering sigma70 promoters based on combining multiple features
Source: BMC Syst Biol. 2018 Apr 24;12(Suppl 4):44. doi: 10.1186/s12918-018-0570-1 (PMC5998878; doi:10.1186/s12918-018-0570-1)
Supplement: Supplementary file 4 — Table S3. Comparison prediction results of different nTrees. (DOC 42 kb) [file 12918_2018_570_MOESM4_ESM.doc]

**Table S3 Comparison prediction results of different nTrees**

| Random Forest (nTree) | Sn (%) | Sp (%) | Acc (%) | MCC |
| --- | --- | --- | --- | --- |
| Random Forest (50) | 85.02 | 97.43 | 93.13 | 0.8473 |
| Random Forest (100) | 85.29 | 97.43 | 93.23 | 0.8494 |
| **Random Forest (200)** | **85.29** | **97.79** | **93.46** | **0.8548** |
